# Supplementary material for: UPR-induced intracellular C5aR1 promotes adaptation to the hypoxic tumour microenvironment
Source: Cell Death Dis. 2025 Jul 22;16(1):547. doi: 10.1038/s41419-025-07862-z (PMC12284258; doi:10.1038/s41419-025-07862-z)
Supplement: Supplementary file 1 — Supplemental text [file 41419_2025_7862_MOESM1_ESM.docx]

**Supplementary Information**

**Supplementary Fig. 1. C5aR1 is highly expressed in hypoxic tumour regions**

For the whole figure: Individual biological replicates (large points) represent the average of the technical replicates (small points). p values were calculated using biological replicates by two-tailed paired Student’s t test (B-D, and F) and one-way ANOVA with Dunnett’s test (E).

**(A)** Pearson’s correlation between C5aR1 mRNA expression and Ahmed Hypoxia signature in TCGA ovarian (left) and prostate (right) cancer samples. *R* score and p value are shown.

**(B-D)** RKO (B), SKOV3 (C), and PC3 (D) cells were cultured under normoxia or hypoxia (<0.1% O_2_) for 24 hours (hr) and subjected to qRT-PCR. n=3.

**(E and F)** C5 mRNA levels in the experiments of Figure 1D and 1E (E), and Figure S1C and S1D (F) were shown. n=3.

**Supplementary Fig. 2. ER stress induces C5aR1 expression in cancer cells under hypoxia (<0.1% O_2_)**

For the whole figure: Individual biological replicates (large points) represent the average of the technical replicates (small points). p values were calculated using biological replicates by two-tailed paired Student’s t test (B, C and E-G), two-tailed unpaired Student’s t test (I and J), two-way ANOVA with uncorrected Fisher’s LSD test (K-O), or one-way ANOVA with Dunnett’s test (D).

**(A)** HIF-1α- KO and WT HCT116 cells were cultured under normoxia or hypoxia (<0.1% O_2_) for 24 hr, and subjected to immunoblotting.

**(B)** CA9 mRNA levels in the experiments of Figure 2B are shown. n=3.

**(C)** p53- KO HCT116 cells were cultured under normoxia or hypoxia (<0.1% O_2_) for 24 hr, and subjected to qRT-PCR. n=3.

**(D)** HCT116 cells were cultured in the indicated conditions for 24 hr, and subjected to qRT-PCR. n=3.

**(E-J)** HCT116 cells were cultured in the same conditions as Figures 2B-D, and subjected to qRT-PCR (E-G, I and J) and immunoblotting (H). n=3.

**(K)** XBP1s (downstream gene of IRE1) mRNA levels in the experiments of Figure 2E are shown. n=3.

**(L)** CHOP and XBP1s (downstream genes of PERK) mRNA levels in the experiments of Figure 2F are shown. n=3.

**(M)** HERPUD1 and ERO1B (downstream genes of ATF6) mRNA levels in the experiments of Figure 2G were shown. n=3.

**(N)** XBP1s, ATF4 and ATF6 mRNA levels in the experiments of Figure 2H are evaluated to analyse knockdown efficiency. n=4.

**(O)** XBP1s, ATF4 and ATF6 mRNA levels in the experiments of Figure 2I are evaluated to analyse knockdown efficiency. n=3

**(P)** Pearson’s correlation of C5aR1 mRNA expression and Xhu UPR signature in TCGA ovarian (left) and prostate (right) cancer samples. *R* score and p value are shown.

**(Q)** Pearson’s correlation between Xhu UPR and Ahmed Hypoxia signature in TCGA colorectal cancer samples. *R* score and p value are shown.

**Supplementary Fig. 3. Hypoxia-induced C5aR1 mediates cellular adaptation to hypoxic stress by regulating cancer cell death**

For the whole figure: Individual biological replicates (large points) represent the average of the technical replicates (small points)*.* p values were calculated using biological replicates by two-way ANOVA with uncorrected Fisher’s LSD test (A, B, D, F, G, H, J and K), two-tailed paired Student’s t test (E and L), or two-way ANOVA with Sidak’s multiple comparison test (I).

**(A and B)** HCT116 cells were transfected with either siRNA against C5aR1 (siC5aR1) or scramble siRNA (siScr) for negative control, cultured under normoxia or hypoxia (<0.1% O_2_) for 24 hr, and subjected to qRT-PCR (A) and Flow Cytometry following permeabilisation (B). n=3.

**(C)** RKO cells were transfected with either pcDNA3.1/C5aR1-GFP (C5aR1) or its empty vector (EV), cultured under normoxia or hypoxia (<0.1% O_2_) for 24 hr, and subjected to immunocytochemistry. C5aR1 (red) or DAPI (blue). Scale bar=10 µm.

**(D)** HCT116 cells were transfected with either pcDNA3.1/C5aR1 (C5aR1) or its empty vector (EV), cultured under normoxia or hypoxia (<0.1% O_2_) for 24 hr, and subjected to qRT-PCR. n=3.

**(E)** LN229 cells were transfected with either siC5aR1 or siScr, cultured under normoxia for 72 hr and subjected to qRT-PCR. n=3.

**(F)** LN229 cells were transfected with either siC5aR1 or siScr, cultured under normoxia or hypoxia (<0.1% O_2_) for 16 hr and subjected to apoptosis assay. n=3.

**(G and H)** HCT116 (G) and RKO (H) cells were transfected with siRNA against C5 (siC5) or siScr, cultured under normoxia or hypoxia (<0.1% O_2_) for 24 hr, and subjected to qRT-PCR. n=3.

**(I)** HCT116 cells were transfected with either siC5aR1 or siScr, cultured under normoxia or hypoxia (<0.1% O_2_) for 16 hr, and subjected to FACS for cell cycle analysis. n=3.

**(J and K)** After transfection with either siC5aR1 or siScr, HCT116 cells were subjected to immunocytochemistry for γH2AX and RPA32 foci assay following hypoxic treatment for 24 and 8 hr, respectively. n=3.

**(L)** LN229 cells were transfected with either siC5aR1 or siScr, cultured under normoxia or hypoxia (<0.1% O_2_) for 16 hr and subjected to clonogenic survival assay. n=3.

**Supplementary Fig. 4. Pharmacologically targeting C5aR1 results in reduced tumour cell survival with enhanced autophagy and apoptosis**

For the whole figure: Individual biological replicates (large points) represent the average of the technical replicates (small points)*.* p values were calculated using biological replicates by one-way ANOVA with Dunnett’s test (A and B), one-way ANOVA with Sidak test (C, F, and I), two-way ANOVA with Tukey test (E and H), and Shapiro-Wilk test and Lognormality test (D, G, and J).

**(A and B)** HCT116 cells were pretreated for 8 hr with the indicated dose of C5aR1 antagonists/inhibitors, PMX205 (A), JPE-1375 and Avacopan (B), cultured under normoxia or hypoxia (<0.1% O_2_) for 24 hr, then cultured under normoxia for 40 hr, and subjected to cell viability assays. n=3.

**(C)** For Fig. 4F, changes in diameter of each spheroid were calculated using a single regression analysis.

**(D)** For Fig. 4F, normality was assessed and confirmed.

**(E-J)** Experiments to confirm the reproducibility of Figure 4F. Diameters of HCT116 spheroids were measured daily after the administration of the indicated drug. Changes in diameter of spheroids (E and H), changes in diameter of each spheroid calculated using a single regression analysis (F and I), and normality test (G and J) are shown. n=11-12

**Supplementary Fig. 5. Regulation of intracellular C5aR1 pools following hypoxia-mediated endocytosis**

For the whole figure: Individual biological replicates (large points) represent the average of the technical replicates (small points)*.* p values were calculated using biological replicates by two-tailed paired Student’s t test (A, B, and H).

**(A and B)** LN229 (A) and HCT116 (B) cells were cultured under normoxia or hypoxia (<0.1% O_2_) for 16 hr and 24 hr, respectively, and subjected to FACS, with (right) or without (left) permeabilisation. n=3.

**(C-G)** RKO cells were cultured under normoxia or hypoxia (<0.1% O_2_) for 24 hr, and subjected to immunofluorescence. C5aR1 (green), DAPI (blue), or organelle makers (red); LAMP for lysosome (C), AIF for mitochondria (D), PDI for endoplasmic reticulum (E), EEA1 for endosomal membrane (F), RCAS1 for Golgi (G). Scale bar=10 µm.

**(H)** HCT116 cells were treated with 2 µM thapsigargin (Thap) or its vehicle (DMSO) for 16 hr and then subjected to FACS with (lower) or without (upper) permeabilisation. n=3.

**(I)** HCT116 cells were cultured under normoxia or hypoxia (<0.1% O_2_) for 4 hr in the presence of 100 µM Dynasore and transferrin conjugated with Alexa Fluor 594, and then transferrin localisation was assessed by immunofluorescence.

**Supplementary Table 1.** List of primers used in qRT-PCR experiments.

| **target gene** | **forward primer (5’-3’)** | **reverse primer (5’-3’)** |
| --- | --- | --- |
| ACTB | ACATCCGCAAAGACCTCTACG | TTGCTGATCCACATCTGCTGG |
| 18S | GTGGAGCGATTTGTCTGGTT | ACGCTGAGCCAGTCAGTGTA |
| C5aR1 | TCCTTCAATTATACCACCCCTGA | ACGCAGCGTGTTAGAAGTTTTAT |
| CA9 | CTTGGAAGAAATCGCTGAGG | TGGAAGTAGCGGCTGAAGTC |
| C5 | CTCCTCAGGCCATGTTCATT | TCTTTTGGCTGGCTTCAAGT |
| CHOP | GGGAGCTGGAAGCCTGGTA | CCCCCATTTTCATCTGAAGACA |
| XBP1s | TGCTGAGTCCGCAGCAGGTG | GCTGGCAGGCTCTGGGGAAG |
| HERPUD1 | AACGGCATGTTTTGCATCTG | GGGGAAGAAAGGTTCCGAAG |
| ERO1B | AATCTGAAGCGACCTTGTCC | GCCCAGCTTTTATTCCAACC |
| ATF4 | GGAGATAGGAAGCCAGACTACA | GGCTCATACAGATGCCACTATC |
| ATF6 | CAGACAGTACCAACGCTTATGCC | GCAGAACTCCAGGTGCTTGAAG |

**Supplementary Table 2.** List of genes for gene signatures used in TCGA analysis

| ***Ahmed hypoxia signature*** | ***Zhu UPR signature*** |
| --- | --- |
| ANXA1 | LOXL2 |
| CALD1 | CALR |
| CP | DNAJB4 |
| IGFBP2 | THBS1 |
| IGFBP5 | CRYAB |
| LOX | SULT1A3 |
|  | KDELR3 |
|  | HSPB2 |
|  | HSPB7 |
|  | CREB3L3 |
|  | FBXO6 |
|  | HSPB6 |
