# Supplementary figures and images for: UPR-induced intracellular C5aR1 promotes adaptation to the hypoxic tumour microenvironment
Source: Cell Death Dis. 2025 Jul 22;16(1):547. doi: 10.1038/s41419-025-07862-z (PMC12284258; doi:10.1038/s41419-025-07862-z)

**A**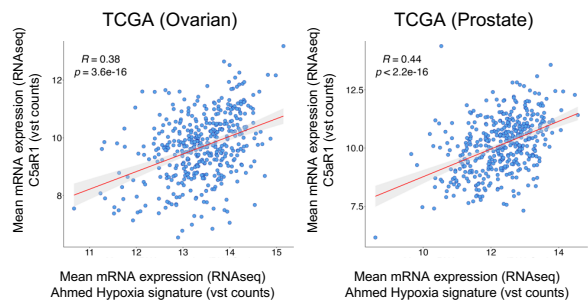**B**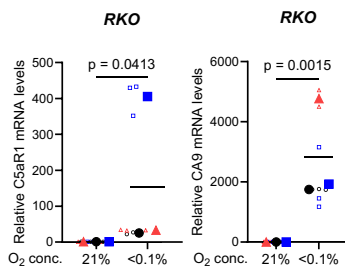**C**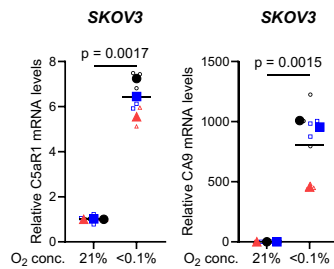**D**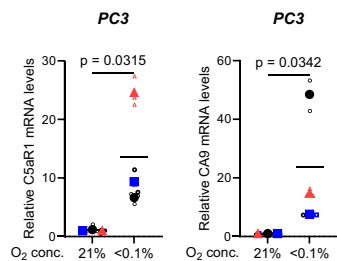**E**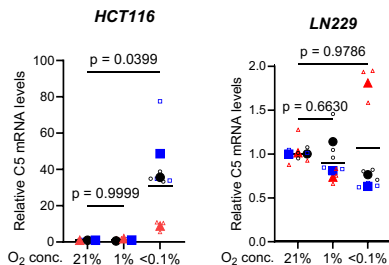**F**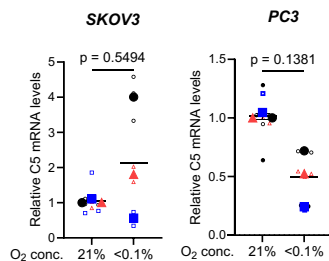

Supplement: Supplementary file 2 — Supplemental Figure 1 [file 41419_2025_7862_MOESM2_ESM.pdf]

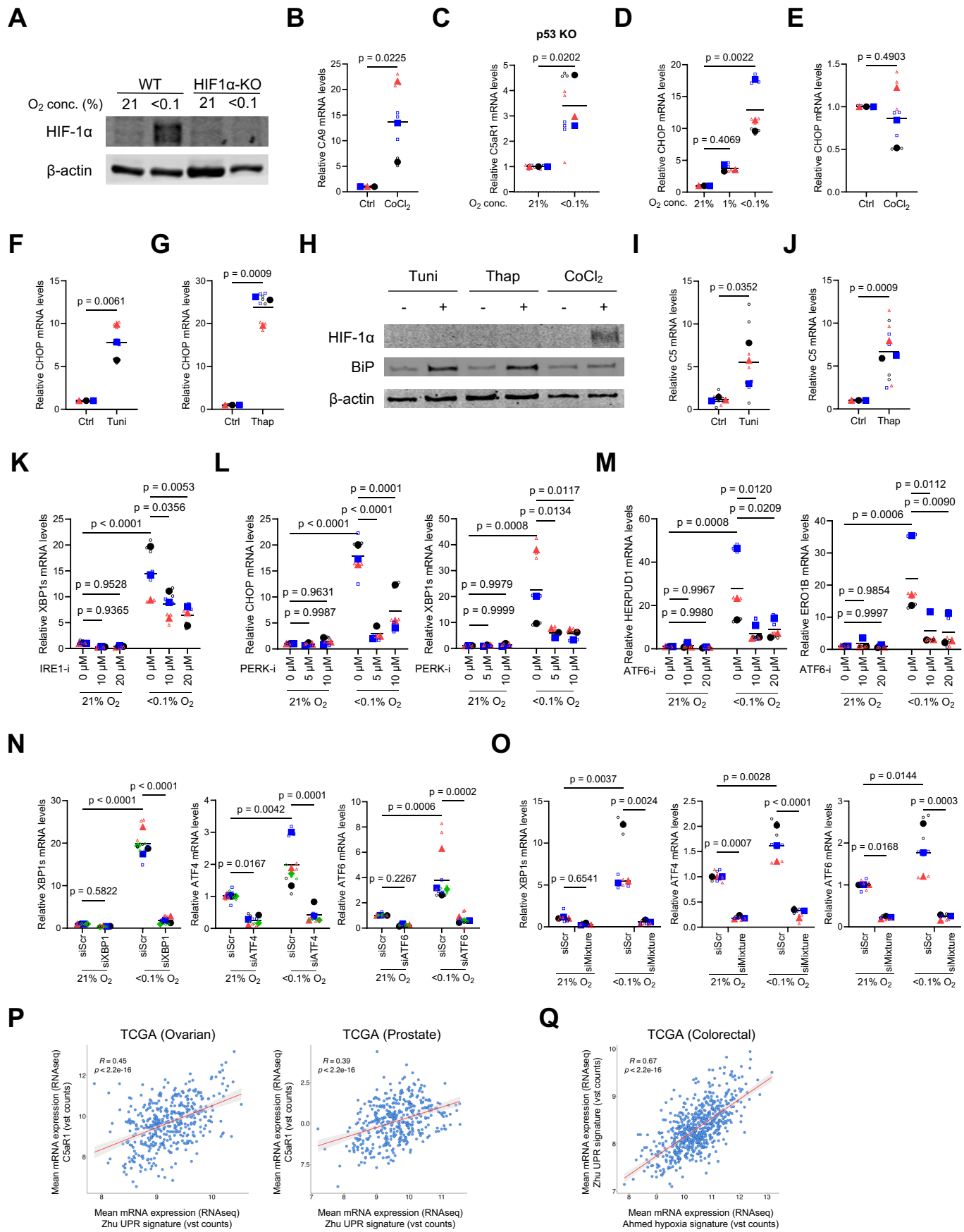

Supplement: Supplementary file 3 — Supplemental Figure 2 [file 41419_2025_7862_MOESM3_ESM.pdf]

A

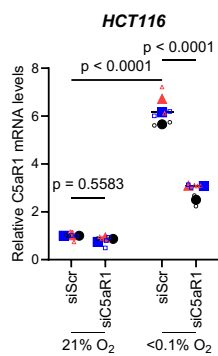

B

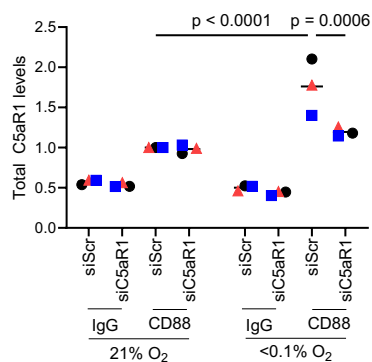

C

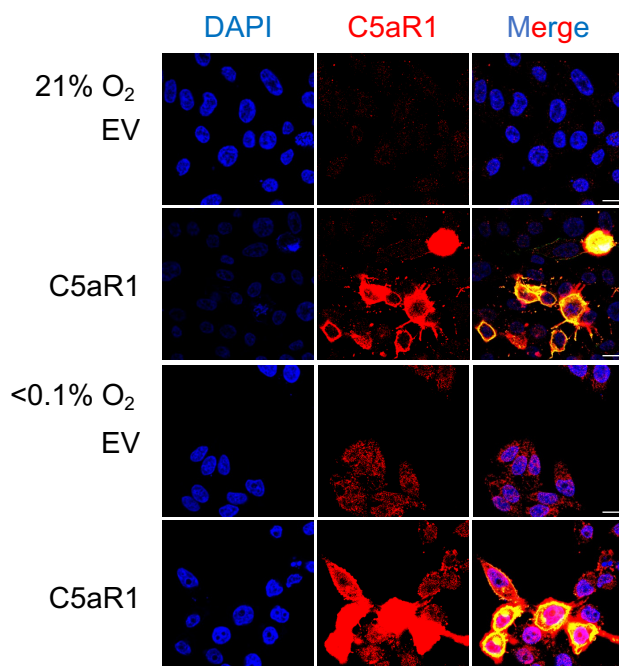

D

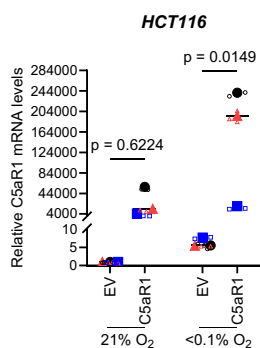

E

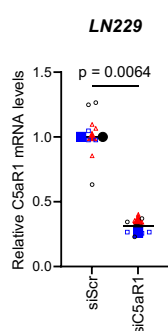

F

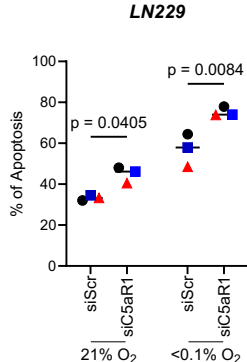

G

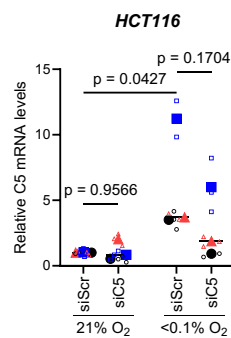

H

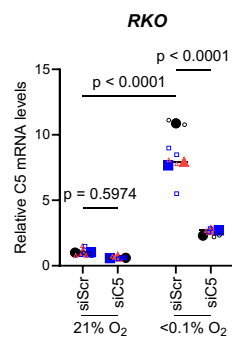

I

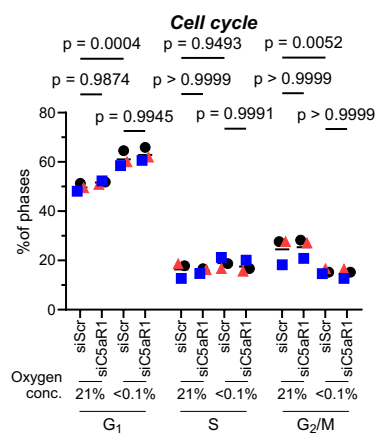

J

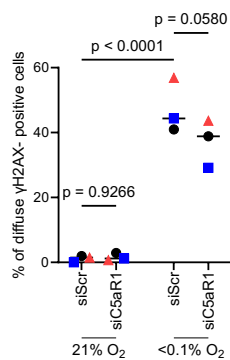

K

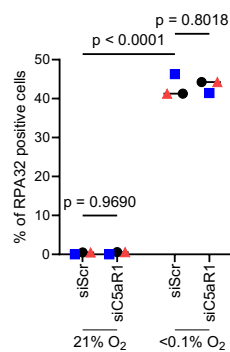

L

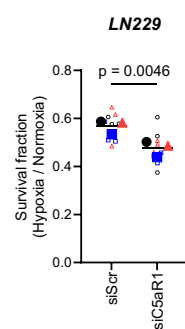

Supplement: Supplementary file 4 — Supplemental Figure 3 [file 41419_2025_7862_MOESM4_ESM.pdf]

**A**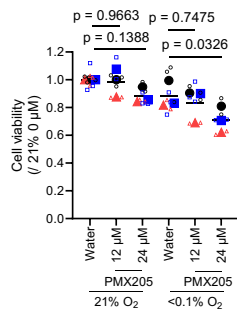**B**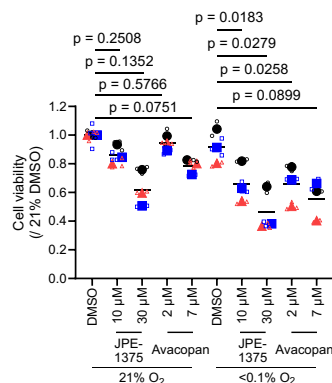**C**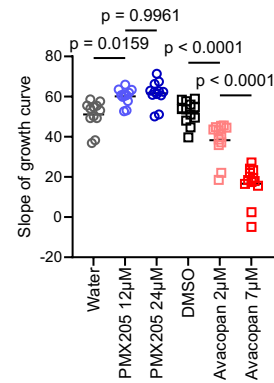**D**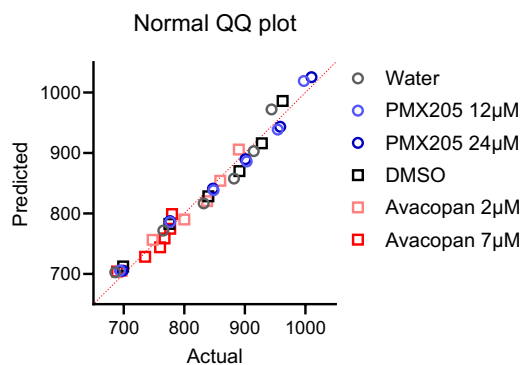**E**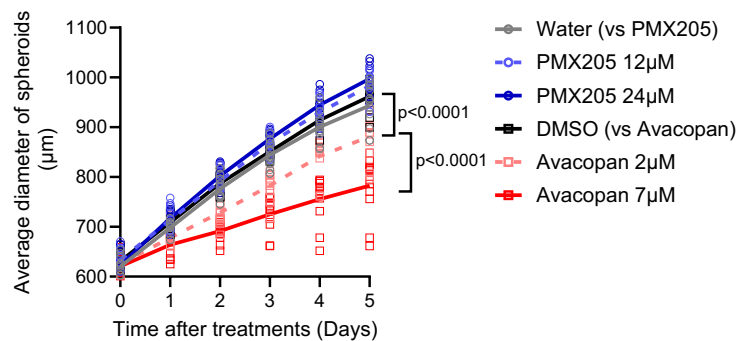**F**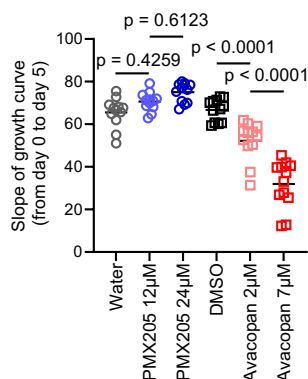**G**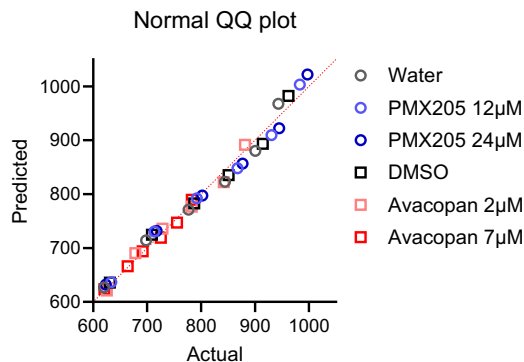**H**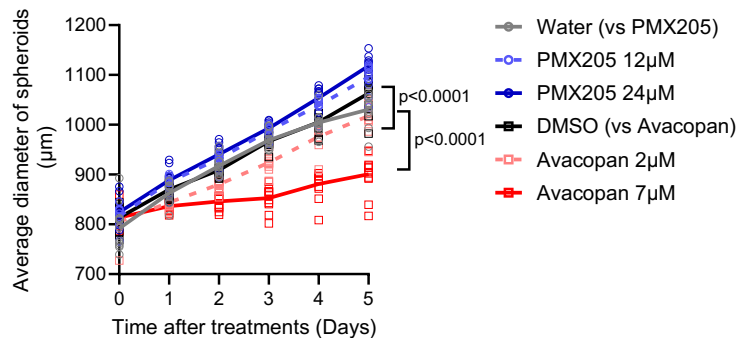**I**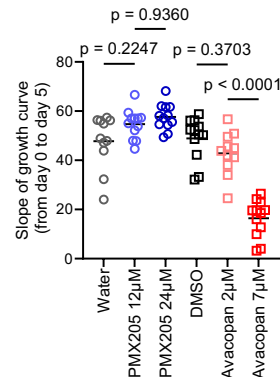**J**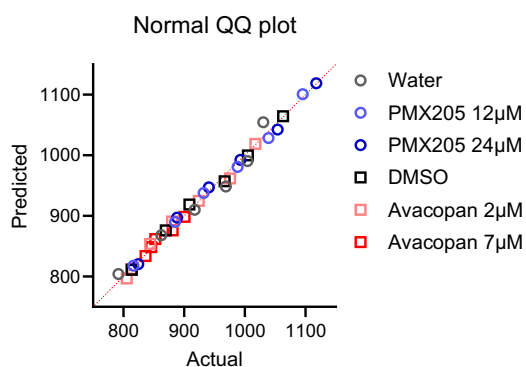

Supplement: Supplementary file 5 — Supplemental Figure 4 [file 41419_2025_7862_MOESM5_ESM.pdf]

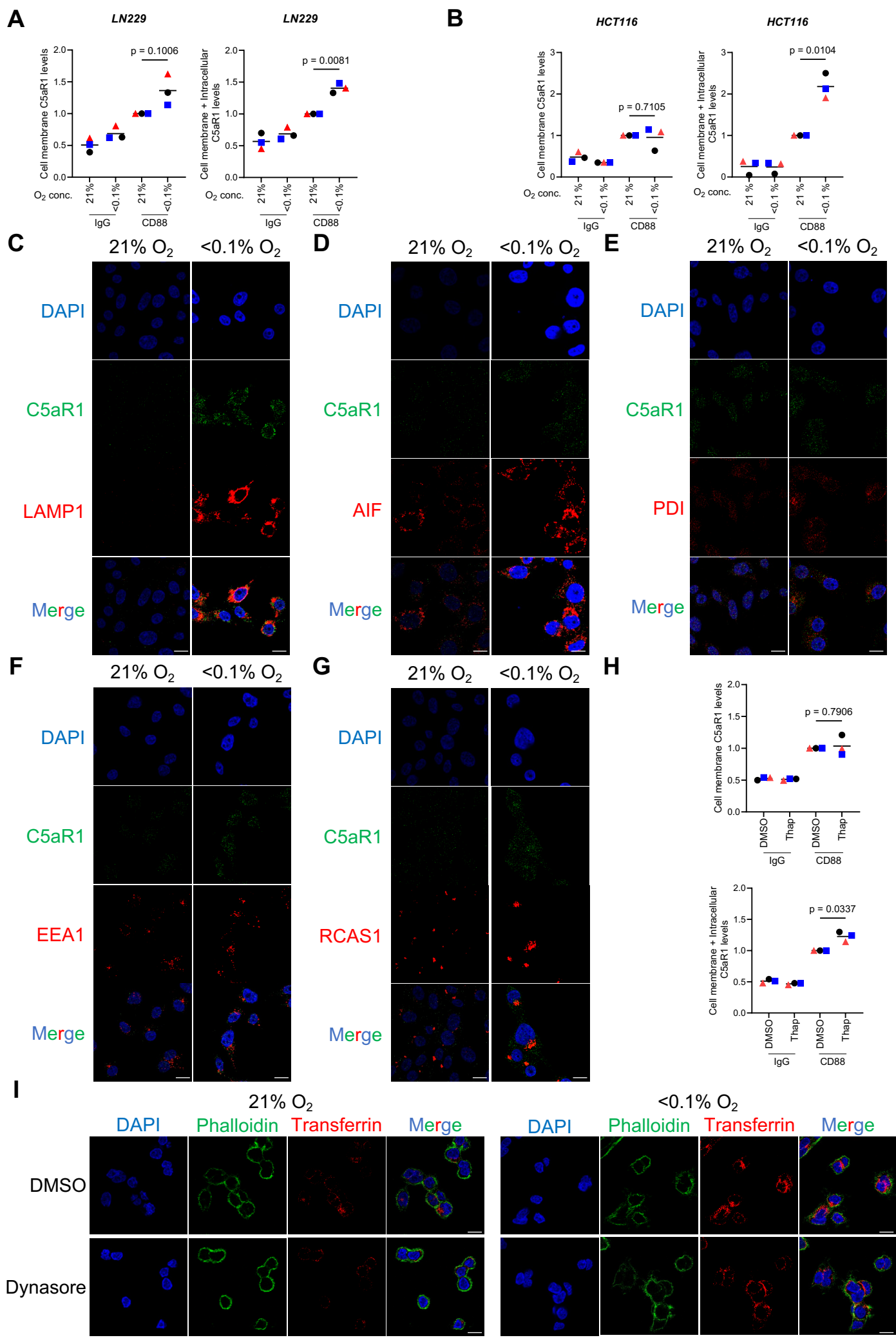

Supplement: Supplementary file 6 — Supplemental Figure 5 [file 41419_2025_7862_MOESM6_ESM.pdf]
