# Supplementary material for: UPR-induced intracellular C5aR1 promotes adaptation to the hypoxic tumour microenvironment
Source: Cell Death Dis. 2025 Jul 22;16(1):547. doi: 10.1038/s41419-025-07862-z (PMC12284258; doi:10.1038/s41419-025-07862-z)
Supplement: Supplementary file 7 — Original Data 1 [file 41419_2025_7862_MOESM7_ESM.pdf]

Full unedited gel for Fig. 3A

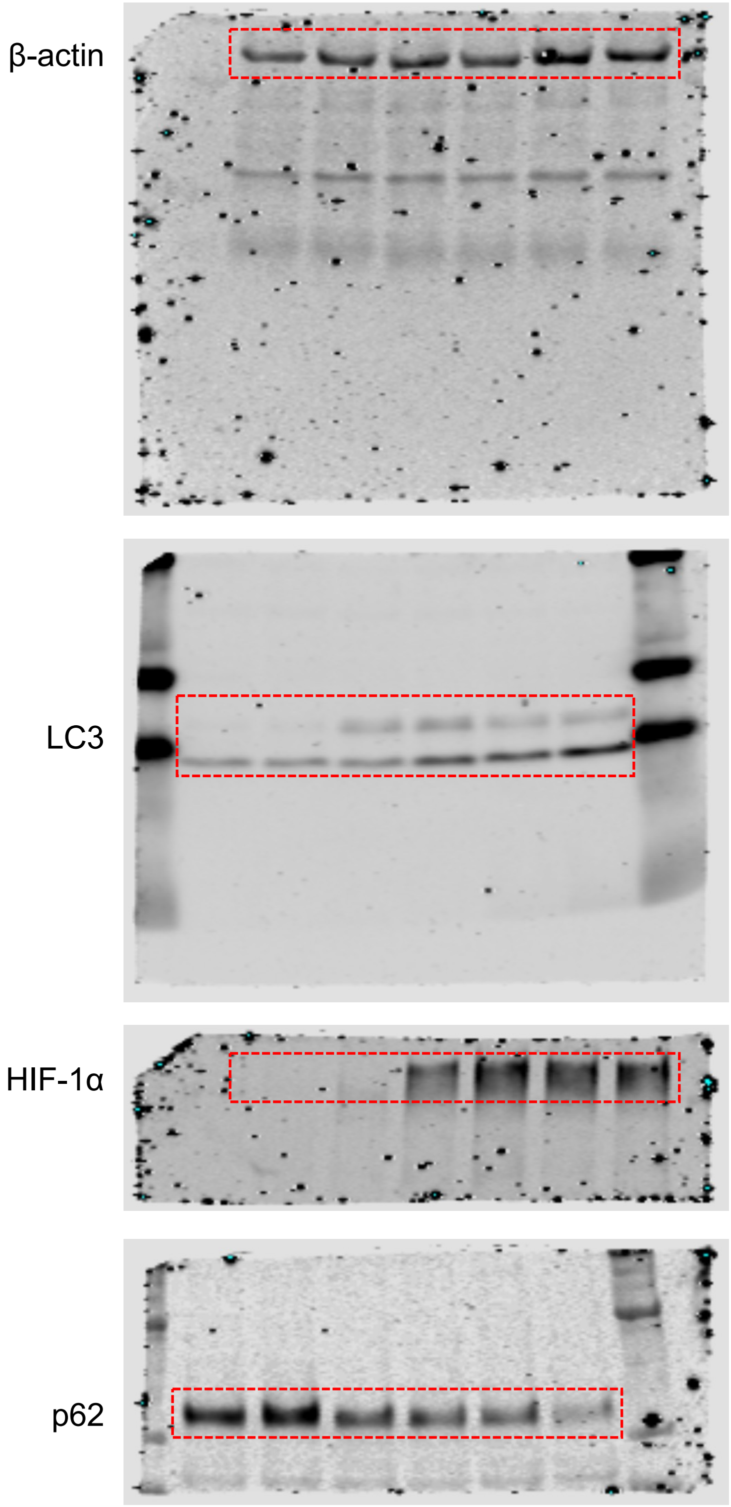

Full unedited gel for Fig. 3B

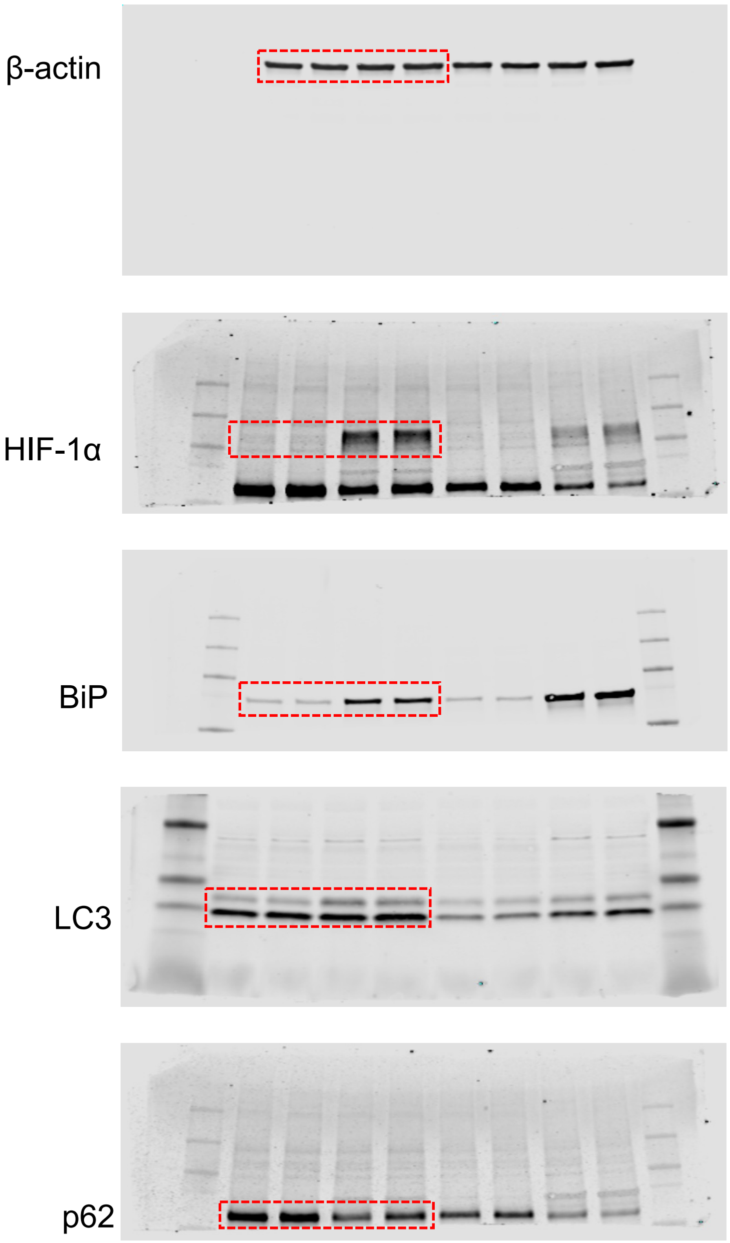

Full unedited gel for Fig. 3E

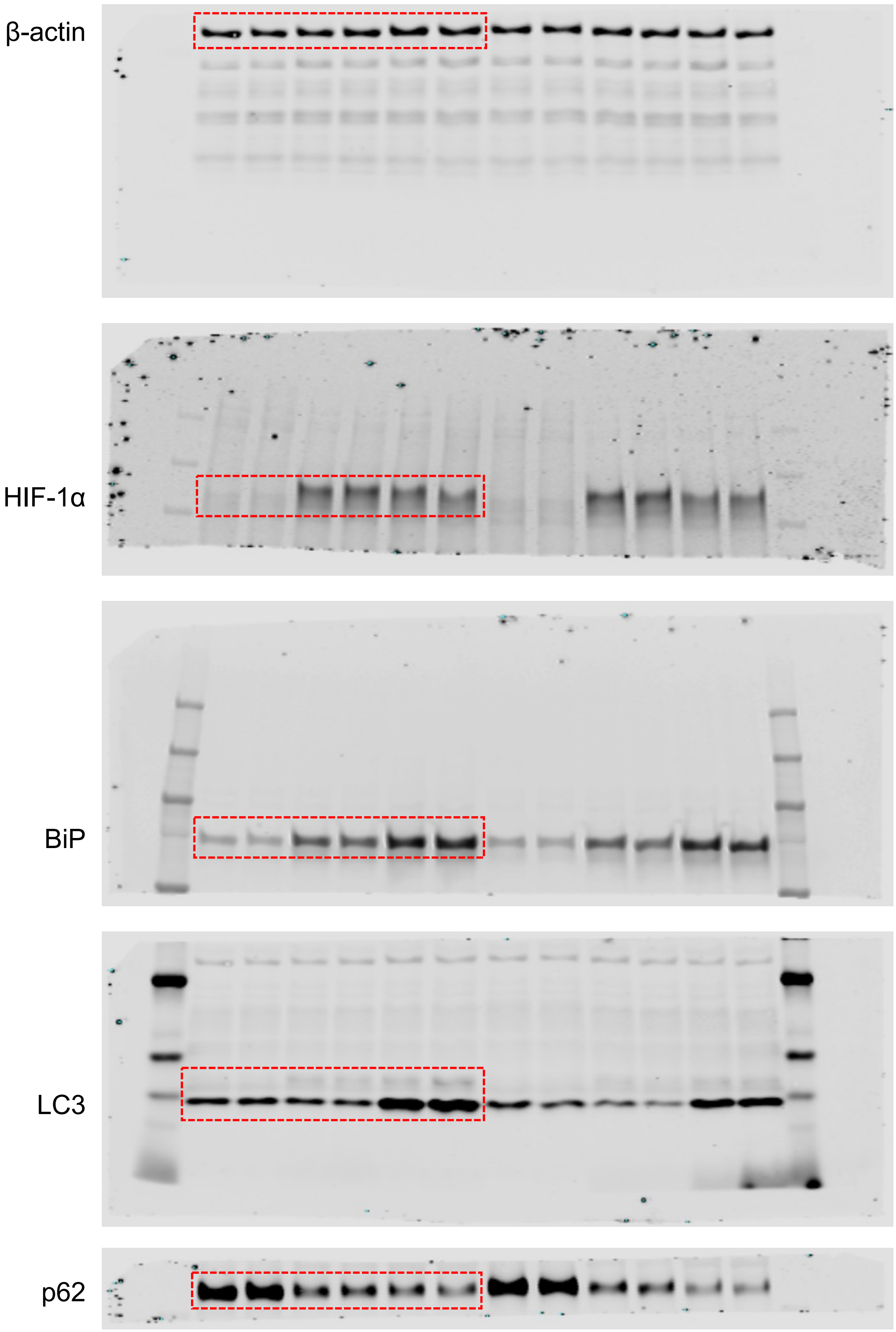

Full unedited gel for Fig. 4D

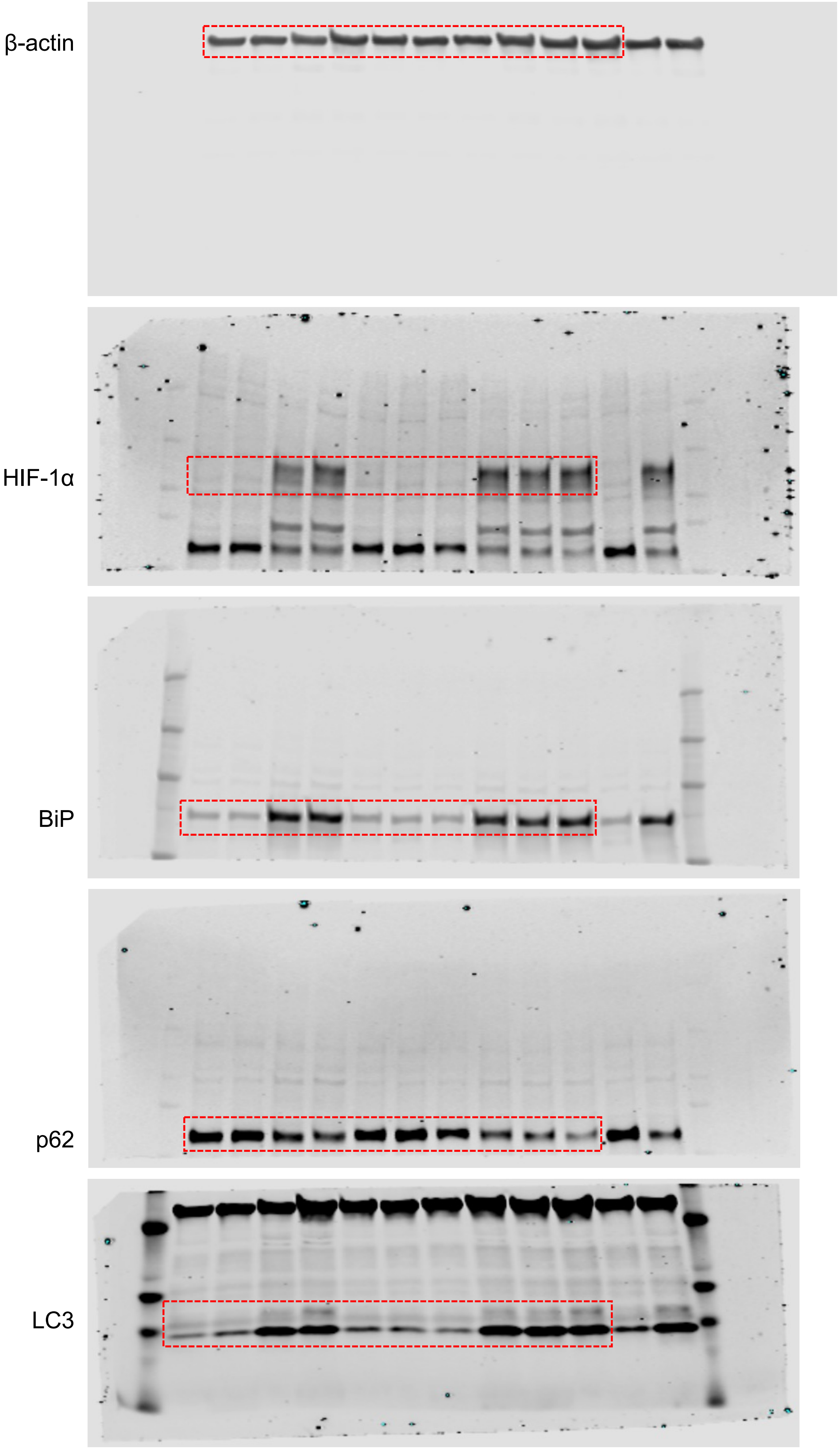

Full unedited gel for Supplementary Fig. 2A

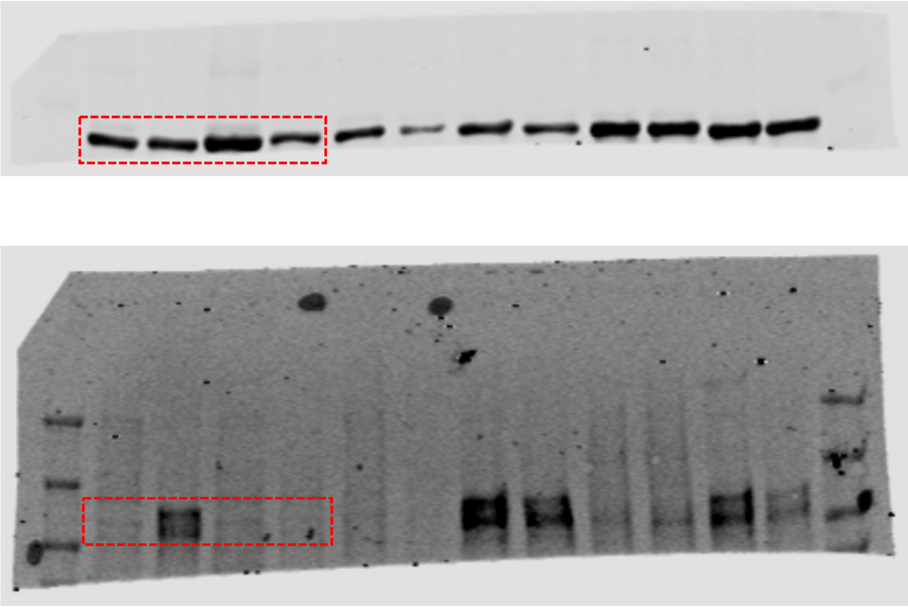

β-actin

HIF-1α

Full unedited gel for Supplementary Fig. 2H

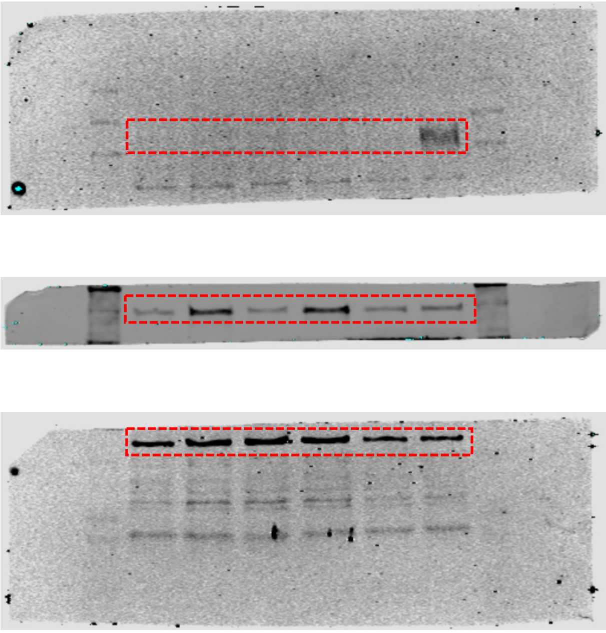

HIF-1α

BiP

β-actin
